# Application of the pMHC Array to Characterise Tumour Antigen Specific T Cell Populations in Leukaemia Patients at Disease Diagnosis

**DOI:** 10.1371/journal.pone.0140483

**Published:** 2015-10-22

**Authors:** Suzanne E. Brooks, Stephanie A. Bonney, Cindy Lee, Amy Publicover, Ghazala Khan, Evelien L. Smits, Dagmar Sigurdardottir, Matthew Arno, Demin Li, Ken I. Mills, Karen Pulford, Alison H. Banham, Viggo van Tendeloo, Ghulam J. Mufti, Hans-Georg Rammensee, Tim J. Elliott, Kim H. Orchard, Barbara-ann Guinn

**Affiliations:** 1 Cancer Sciences Unit (MP824), Somers Cancer Sciences Building, University of Southampton, Southampton, United Kingdom; 2 Department of Haematology, Southampton University Hospitals Trust, University of Southampton, Southampton, United Kingdom; 3 Department of Life Sciences, University of Bedfordshire, Park Square, Luton, United Kingdom; 4 Laboratory of Experimental Haematology, Vaccine and Infectious Disease Institute, University of Antwerp, Wilrijkstraat 10, B-2650 Antwerp, Belgium; 5 Department of Immunology, Institute for Cell Biology, University of Tübingen, Tübingen, Germany; 6 King’s Genomics Centre, School of Biomedical and Health Sciences, King's College London, London, United Kingdom; 7 Nuffield Division of Clinical Laboratory Sciences, Radcliffe Department of Medicine, University of Oxford, John Radcliffe Hospital, Oxford, United Kingdom; 8 Blood Cancer Research Group, Centre for Cancer Research and Cell Biology (CCRCB), Queen’s University Belfast, Belfast, United Kingdom; 9 Department of Haematological Medicine, King's College London School of Medicine, London, United Kingdom; University of Pittsburgh, UNITED STATES

## Abstract

Immunotherapy treatments for cancer are becoming increasingly successful, however to further improve our understanding of the T-cell recognition involved in effective responses and to encourage moves towards the development of personalised treatments for leukaemia immunotherapy, precise antigenic targets in individual patients have been identified. Cellular arrays using peptide-MHC (pMHC) tetramers allow the simultaneous detection of different antigen specific T-cell populations naturally circulating in patients and normal donors. We have developed the pMHC array to detect CD8^+^ T-cell populations in leukaemia patients that recognise epitopes within viral antigens (cytomegalovirus (CMV) and influenza (Flu)) and leukaemia antigens (including Per Arnt Sim domain 1 (PASD1), MelanA, Wilms’ Tumour (WT1) and tyrosinase). We show that the pMHC array is at least as sensitive as flow cytometry and has the potential to rapidly identify more than 40 specific T-cell populations in a small sample of T-cells (0.8–1.4 x 10^6^). Fourteen of the twenty-six acute myeloid leukaemia (AML) patients analysed had T cells that recognised tumour antigen epitopes, and eight of these recognised PASD1 epitopes. Other tumour epitopes recognised were MelanA (n = 3), tyrosinase (n = 3) and WT1_126-134_ (n = 1). One of the seven acute lymphocytic leukaemia (ALL) patients analysed had T cells that recognised the MUC1_950-958_ epitope. In the future the pMHC array may be used provide point of care T-cell analyses, predict patient response to conventional therapy and direct personalised immunotherapy for patients.

## Introduction

The outcomes for patients with leukaemia have improved considerably over the last 30 years due to enhancements in supportive care, expertise and the development of haematopietic stem cell transplants. Most patients now achieve a first remission following combinational chemotherapy protocols, but the best treatment option for patients for whom it is applicable remains allo-transplantion, an option which depends on meeting health criteria and donor availability. First remission is expected to be the optimal time-point for immunotherapy to be administered, when residual disease loads are low.

Tumour antigens have been shown to induce specific T-cell responses in patients and thus act as targets for immunotherapy treatments [1–3]. A number of end-point assays have been developed to assess both cellular (Enzyme-Linked ImmunoSpo (ELISpot), cytotoxic T-lymphocyte (CTL) assays) and humoral (enzyme-linked immunosorbent assay (ELISA)) immune responses induced in patients during immunotherapy clinical trials, for use as indicators of clinical efficacy. The development of peptide–MHC tetramers (pMHC) [4] and variants thereof [5,6] have allowed the visualization of antigen-specific T cells through flow cytometry and the use of *in situ* [7] approaches. Such analyses have enabled specific T-cell enumeration, assessment of cytokine production/secretion (flow based assays, ELIspot) and the expansion of T-cell populations for functional analysis (CTL analysis). However flow cytometry is technically difficult, time consuming, expensive and, until recently, limited to only one or a very few antigen specificities per sample [8]. This changed with the description of pMHC microarrays by Soen *et al* in 2003 [9]. Soen *et al* demonstrated the potential of the pMHC arrays to detect OVA-antigen specific populations from both T-cell receptor (TCR) transgenic and wild type mice. Subsequently Chen et al [10] used the pMHC arrays to detect multiple T-cell populations in 11 human leukocyte antigen (HLA)-A*0201 positive patients with resected stage IIC/III and IV melanoma who were undergoing a melanoma-associated peptide vaccine trial. In addition to showing the presence of specific T-cell populations that could recognise epitopes within independent antigens, the authors demonstrated functionality through the co-spotting of pMHCs with a panel of cytokines.

Similar methods such as the Fluorescence-activated cell sorting (FACS)-based “combinatorial encoding” approach [8,11], despite being elegant and robust, are limited in the number of T-cell populations they can detect (25 and ≤15 different T-cell populations in a single sample, respectively). Hadrup et al [8] suggested that the analysis of more than 100 T-cell populations per sample may be possible in the future through the use of a larger number of fluorochromes and multi-dimensional combinatorial encoding while Newell et al [11] stated that 31 and 63 populations could be analysed with five- and six-colour FACs. Recently Newell et al [12] demonstrated that through combining combinatorial [8,11] and mass cytometry–based pMHC staining approaches [13] that they could detect more than 100 specific T-cell populations as well as 20–30 phenotypic markers, in a single blood or intestinal lymphocyte sample. However combinatorial approaches require expensive reagents (quantum dots [8]) and/or complex multifactorial analysis, with four or more multi-laser FACS analysis being essential.

We have developed a pMHC array to analyse T cells from leukaemia patients to determine epitope-specific recognition from a range of cancer-testis (CT) and leukaemia-associated antigens (LAAs). In contrast with previous studies, these samples were taken either at disease presentation prior to treatment or at relapse following conventional therapies and none of the samples were stimulated to expand specific T-cell populations prior to pMHC array analysis.

## Materials and Methods

### Patient samples

All research involving human participants was approved by the National Research Ethics Committee South Central (REC Reference 07/H0606/88), and all clinical investigation were conducted according to the principles expressed in the Declaration of Helsinki. Written informed consent was obtained from every participant. Normal donor buffy coats were obtained from the National Blood Service UK or patient samples (26 AML, five chronic myeloid leukaemia (CML) and seven ALL)(S1 Table) and healthy donor blood were obtained from the Department of Haematology, Southampton General Hospital following informed consent (Local Research Ethics Committee, Southampton University Hospitals NHS Trust, Southampton U.K., LREC submission number 228/02/T). Red blood cells were lysed using red cell lysis buffer (15.5mM ammonium chloride (NH_4_Cl), 1mM potassium bicarbonate, 0.01mM EDTA pH8.0). If dead cells exceeded 40% of the total cell number then they were positively removed using the Miltenyi dead cell removal kit and CD8^+^ T cells were negatively isolated from using EasySep CD8 T-Cell Enrichment kit (containing beads to deplete CD4, CD14, CD16, CD19, CD20, CD36, CD56, CD123, GlyA, T cell receptor (TCR)-gd) with CD11b Depletion (EasySep) to consistently provide ≥97% purity of “untouched” CD8^+^ T cells as determined by flow cytometry. All cells which were not used immediately were stored in EX-VIVO 15^TM^ at a final cell number of 10^7^cells/ml (BioWhittacker^TM^, Cambrex,) in the presence of 1% human AB sera and 10% DMSO (both Sigma). CMV serology was determined by standard techniques.

### pMHC molecules

Peptides for MHC multimers and functional assays were synthesised using standard Fmoc chemistry ([4];http://www.syfpeithi.de/) and used as described previously [14]. Biotinylated recombinant MHC peptide monomers, using peptides with well-documented MHC binding on www.syfpeithi.de, were produced essentially as described previously [15]. Briefly, fluorescent multimers were freshly generated by co-incubating biotinylated HLA peptide monomers with 95% streptavidin: 5% streptavidin-AF532 (Molecular Probes, Leiden, the Netherlands) for the pMHC arrays and streptavidin-phycoerythrin (SA-PE) for flow cytometry at a 4:1 molar ratio.

### pMHC arrays

**Fig 1:**
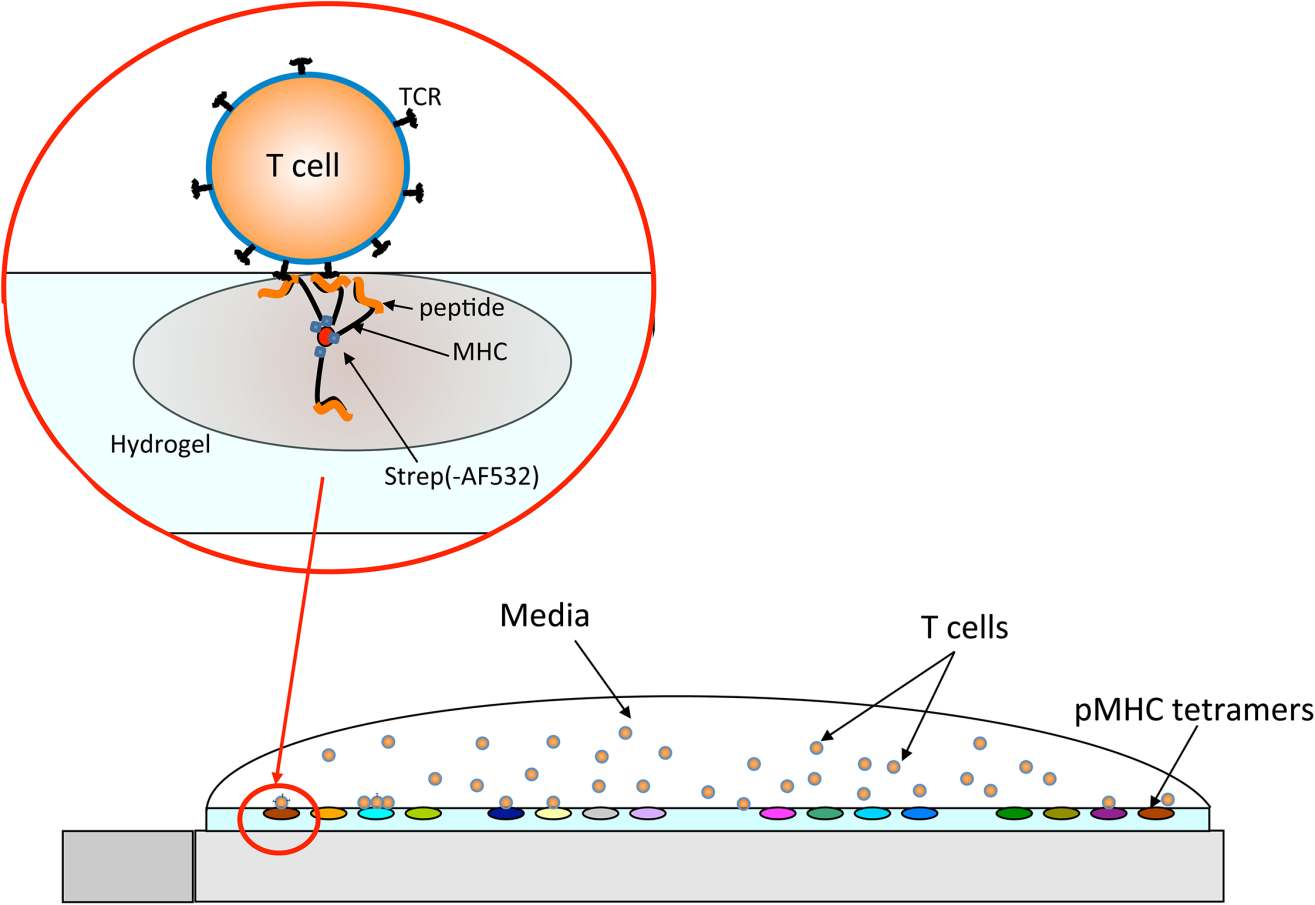
Diagrammatic representation of the pMHC array. Lipophilically dyed CD8^+^ T cells (0.8–1.2 x 10^6^ per microarray) were incubated at 37°C in warm colourless X-VIVO 15 media with the pMHC array. Each pMHC spot contains 1ng of tetramer and each slide can hold up to 1,000 spots of pMHC.

Hydrogel slides were prepared in-house as described previously [16]. pMHC tetramers (**Table 1**) were spotted onto hydrogel slides using a contact deposition-type QArray^2^ printer and HPLF 0.3mm solid tip pins (Genetix), at a concentration of 0.5mg/ml in PBS and 2% glycerol. Approximately 1ng of pMHC tetramer was spotted at each touch point on the hydrogel. Printed arrays were immobilised at 4°C in enhanced humidity (75%) achieved through the co-incubation of slides with wet NaCl in sectioned sealed boxes, for 48 hours. Excess moisture was removed from the non-gel surfaces of the slides and the arrays stored in a sealed box at 4°C prior to use. The selected array was warmed to room temperature. 10^6^/ml CD8^+^ T cells were labelled using a DiD lipophilic tracer (Molecular Probes) according to manufacturer’s instructions and the cells washed three times in colourless X-VIVO 15 (BioWhittacker). Cells were resuspended at 2.5 x 10^6^/ml in colourless X-VIVO 15 and 400μl incubated for 20min at 37°C with the pMHC array (**Fig 1**). Unbound cells were removed by aggressive flicking of the slide, followed by two washes with 10ml warm colourless X-VIVO 15. Excess culture medium was removed before slides were analysed on the ProScanArray (PerkinElmer). Negative controls of AF532 in printing media and random library tetramers [17] were also included. All pMHC spots were printed in blocks of 3 x 3 or 6 spots as part of 1 or 2 consecutive rows and the whole pMHC array of spots was repeated at two independent sites on each hydrogel slide. Hydrogel slides were analysed after incubation with patient samples in the ProScanAarray (Perkin Elmer) to detect binding of labelled T cells to spotted pMHCs.

**Table 1:** pMHCs used on the array to detect virus and LAA-specific T cell populations within the peripheral blood CD8+ population of leukaemia patients.

Epitope	HLA type	Amino acid sequence	Ref^a^	Epitope	HLA type	Amino acid sequence	Ref^a^
ALK-SLA human	HLA-A*0201	SLAMLDLLHV	[18]	p68 RNA-helicase	HLA-A*0201	YLLPAIVHI	[19]
CMV pp65	HLA-A*0201	NLVPMVATV	[20]	Survivin_5-11_	HLA-A*0201	TLPPAWQPL	[21]
Flu M1	HLA-A*0201	GILGFVFTL	[22]	Survivin_96-104_	HLA-A*0201	LTTLGEFLKL	[21]
G250	HLA-A*0201	HLSTAFARV	[23]	Tyrosinase	HLA-A*0201	YMDGTMSQV	[24]
Gp100	HLA-A*0201	KTWGQYWQV	[25]	VMSA_HPBV	HLA-A*0201	WLSLLVPFV	[26]
HAGE	HLA-A*0201	DLILGNISV	[27]	WT1_37-45_	HLA-A*0201	VLDFAPPGA	[28]
HBV	HLA-A*0201	FLLTRILTI	[29]	WT1_126-134_	HLA-A*0201	RMFPNAPYL	[30]
HPV16 E7	HLA-A*0201	YMLDLQPETT	[31]	EBV_BMRF1_105-114_	HLA-A*0101 mut	AVEQASLQFY	NK
HPV16 L1	HLA-A*0201	ICWGNQLFV	[5]	EBV_BZLF_340-348_	HLA-A*0101 mut	VVETLSSSY	NK
Library	HLA-A*0201	^c^	[17]	EBV BZLF	HLA-A*0101 245V^b^	DSELEIKRY	NK
MelanA mod	HLA-A*0201	ELAGIGILTV	[32]	LMP2_EBV_410-420_	HLA-A*0101 mut	LTEWGSGNRTY	NK
MUC1	HLA-A*0201	NLTISDVSV	[33]	MAGE 1_161−169_	HLA-A*0101	EADPTGHSY	[34]
Muc1 mod	HLA-A*0201	KLLLTVLTV	[35]	EBV BMLF1_298-306_	HLA A*0301 mut	SLSKVILTLK	NK
MUC-1 tandem repeat	HLA-A*0201	STAPPVHNV	[36]	EBV BRLF1_148-156_	HLA-A*0301 mut	RVRAYTYSK	[37]
MUC1_HUMAN mod	HLA-A*0201	SLAPPVHNV	[38]	EBV EBNA3_471-479_	HLA-A*0301	RLRAEAQVK	[39]
PASD1(1)	HLA-A*0201	QLLDGFMITL	[40]	LMP1_EBV mod.	HLA-A*0301	ALFLGIVLK	NK
PASD1(2)	HLA-A*0201	YLVGNVCIL	[40]	p53	HLA-A*0301	RVRAMAIYK	[41]
PASD1 (Pa14)	HLA-A*0201	RLWQELSDI	[42]	p53_321-330_	HLA-B*0702	KPLDGEYFTL	[43]
PASD1(5)	HLA-A*0201	ELSDSLGPV	[40]	EBV BZLF1	HLA-B*0801	RAKFKQLL	[44]
Proteinase 3	HLA-A*0201	VLQELNVTV	[45]	EBV EBNA3_325-333_	HLA-B*0801 wt	FLRGRAYGL	[46]

^a^Original reference for the epitope

^b^245V mutation of MHC class I as described by [30]

^c^a random selection of 6,000 peptides, generated as described in reference [17].

HLA: human leukocyte antigen; Mod.: modified; Mut.: mutated; NK: not known.

### Optimisation of the pMHC array

Cut-off points for scoring were decided following the analysis of normal donor CD8+ cells on the pMHC array during the optimisation phase. Although we could look at each spot on the array at high magnification and see individual T cells sticking to spots, we found that when 40 or more cells were bound to a spot we could score this reproducibly as positive at X100 magnification, and when it occurred on three of six spots in two independent regions of the array it concurred with pMHC staining of specific T cells in flow cytometry and CMV-serology data. We reduced background on the pMHC array through aggressive washing and, although background varied somewhat between patient samples, it was easily visualised by virtue of the non-specific sticking of cells and debris outside the area of each pMHC spot. These “dirty” samples were not included in our analyses and we judged acceptable levels of background by a comparison to the patient sample AML006. Every batch of pMHC arrays was tested with the patient sample AML006, which was HLA-A2 negative and negative for CD8+ T cells binding to all pMHCs. It was used to control for within batch stickiness of the arrays outside of the pMHC spots and the sticking of its’ T cells to pMHC spots was always low–no more than 1–2 cells at any one pMHC spot. In addition fluorochrome alone and random library [17] pMHCs were used to provide negative controls for CD8 T cells sticking non-specifically to pMHC spots. The removal of non-CD8 cells from the peripheral blood samples was essential and, as recommended by others [9,10], we always depleted CD11b cells. The pMHC spots were visualised using low concentrations of AF532, minimised to 2.25% of the total streptavidin used to conjugate biotinylated pMHC monomers, to minimise bleeding of AF532 into the DiD channel while allowing the user to co-localise pMHC spots and bound T cells using the ProScanArray.

### Scanning electron microscopy (SEM)

Further analysis of pMHC arrays by SEM required fixing of the hydrogel slides in 3% glutaraldehyde, 4% paraformaldehyde, 0.1M PIPES buffer, pH 7.2, post-fixing in 1% osmium tetroxide, 0.1M PIPES buffer, pH 7.2, dehydration through an ethanol series, critical point dried, mounting on stubs, sputter coated with gold/palladium and subsequent imaging on a Hitachi S800 SEM.

### Flow cytometry

FACS analysis was used to demonstrate HLA-A2 positivity on unselected total white blood cells using the HLA-A2 antibody (clone name: BB7.2) from Serotec. Cells alone and cells incubated with anti-human IgG2a isotype control were also analysed for each sample. The HLA-A2 antibody also detects the 10% of HLA-A2 positive individuals who are HLA-A24 rather than HLA-A*0201 and so a second round of PCR based screening to confirm the full haplotype was performed either by the Anthony Nolan at the Royal Free Hospital or in-house at the Southampton General Hospital. To confirm T-cell populations recognised specific epitopes, we incubated 10^6^ cells with diluted multimers at 4°C for 30min (HLA concentration 5μg/mL) in FACS buffer containing 50% FCS as described previously [47]. Subsequently cells were incubated with anti-CD8-Fluorescein isothiocyanate (FITC) antibodies at 4°C for 20min, washed and analysed on the FACScalibur.

### Immunolabelling

White blood cells from the peripheral blood or bone marrow of patients were defrosted and spotted at 10^7^cells/ml in PBS onto glass slides and allowed to dry for 4-16hrs at room temperature. Cells were then incubated with hybridoma supernatant from the monoclonal antibody PASD1-1 which recognises a region common to the PASD1a and PASD1b proteins (clone 2ALCC136) between aa 195–474 or PASD1-2 (clone 2ALCC128) a monoclonal antibody which is specific for aa 540–773 present only in the longer PASD1b protein [40]. After washing in PBS, the slides were stained using the Mach-Three detection kit (A. Menarini Diagnostics (U.K.), Berkshire) following manufacturer’s instructions. Antigen/antibody complexes were visualized using diaminobenzidine tetrahydrochloride substrate (Sigma).

## Results

### Optimisation of CD8^+^ T cell isolation

**Fig 2:**
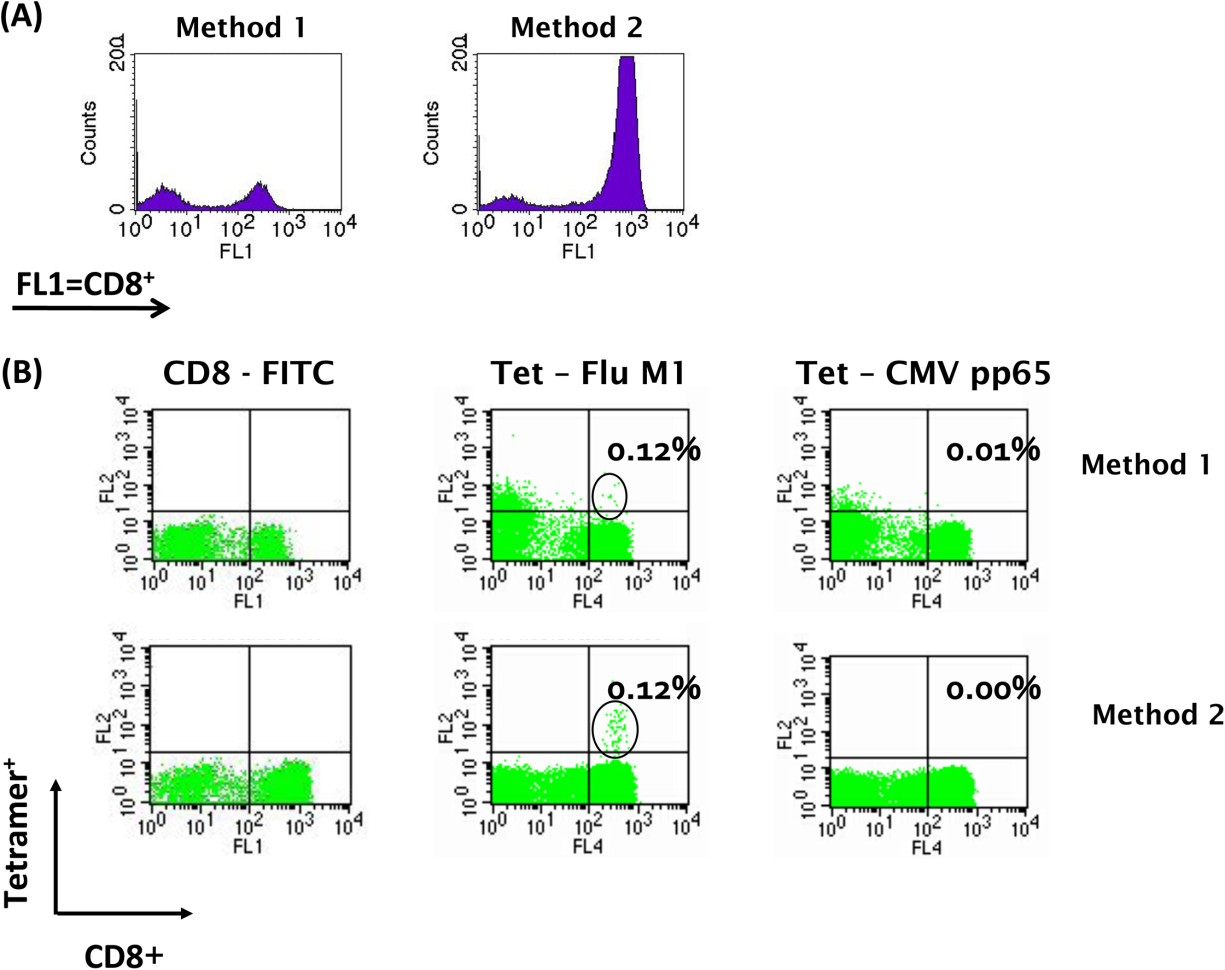
Direct comparison of epitope specific T cell detection by pMHC tetramers following negative CD8^+^ isolation by conventional techniques. CD8^+^ T cells were isolated from HLA-A*0201^+^, normal donor buffy coat using commercially available negative isolation kits. This normal donor was CMV sero-negative, pMHC-CMV pp65 negative and pMHC-Flu M1 positive. **(A)** 10^6^ cells per test were incubated with 1μg of anti-CD8-FITC antibody, the cells washed and analysed by flow cytometry using a FACScalibur^TM^. Method 2 gave the highest number of CD8^+^ cells (FL1). **(B)** 10^6^ CD8 T cells isolated from the same normal donor by Method 1 or 2 were labelled with anti-CD8-FITC (y-axis) and pMHC-(Flu or CMV)-SA-PE (x-axis) and analysed by flow cytometry. We found that Method 2 StemSep negative isolation of CD8s provided the best separation of pMHC-labelled CD8 T cells, in this case with Flu M1 tetramers.

To optimise the negative isolation of CD8^+^ T cells we directly compared two of the most popular techniques using normal donor leukophoresis samples. We found that StemSep beads provided the highest purity of CD8^+^ live cells (**Fig 2A–method 2**) and showed the least dead cells with the most distinct CD8^+^/tetramer^+^ population by flow cytometry (**Fig 2B**) in at least three independent tests.

### Reproducibility of the pMHC array

**Fig 3:**
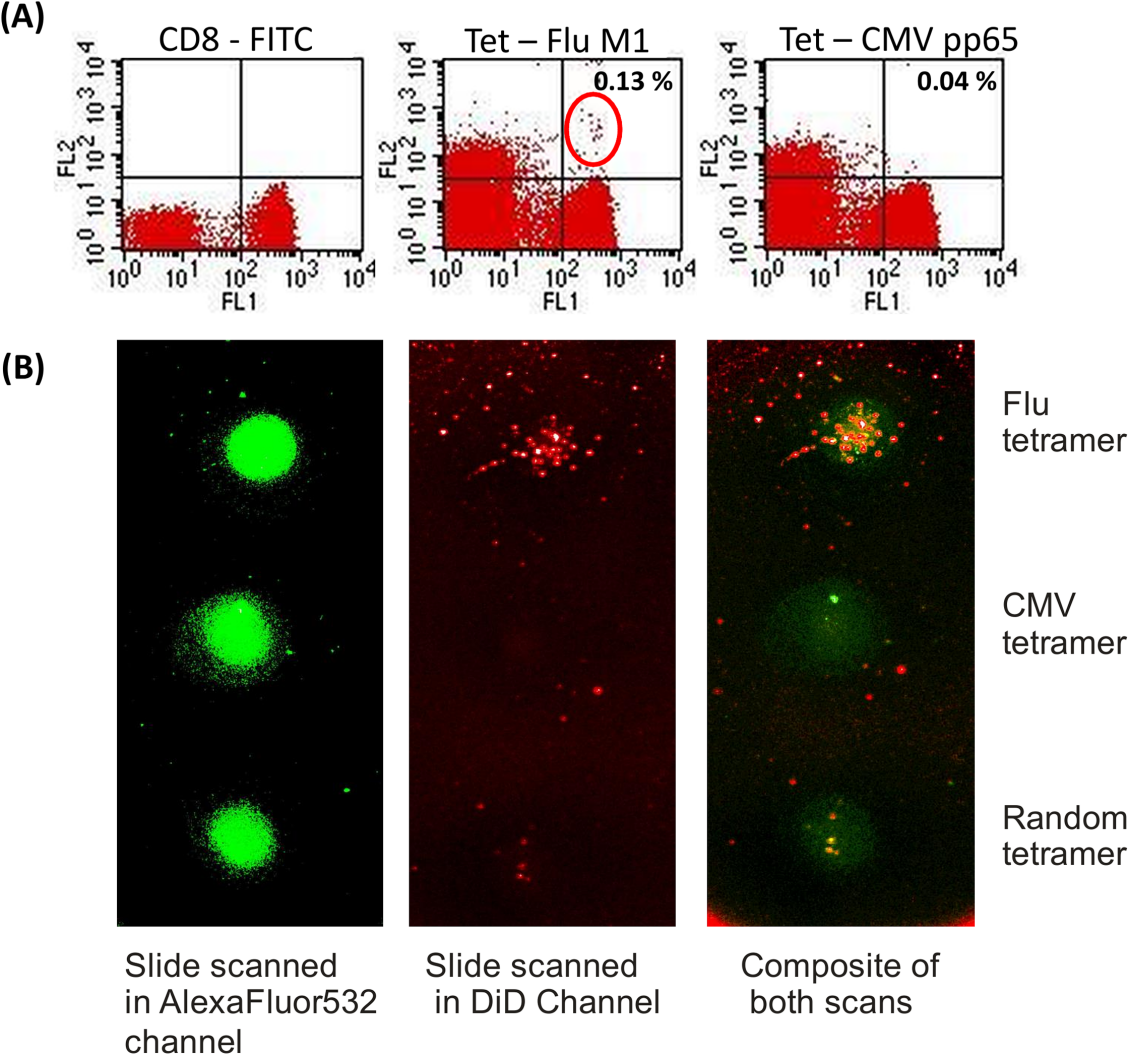
Correlation between the detection of specific T cell populations using flow cytometry and pMHC arrays. Negatively isolated CD8^+^ T cells from a normal donor who was pMHC-Flu M1 positive and CMV sero-negative were **(A)** labelled with anti-CD8-FITC (y-axis) and pMHC-(Flu or CMV)-SA-PE (x-axis) and analysed by flow cytometry. We showed that a minimum 0.7 x 10^6^ CD8^+^ cells (including controls) could be used to show that the sample had Flu M1^+^ specific T cells but not A2/CMV pp65 specific T cells by conventional flow cytometry. CD8-FITC (FL1-H) is shown on the x-axis and pMHC-SA-PE (FL2-H) staining on the y-axis. **(B)** 10^6^ CD8^+^ T cells/ml, from the same normal donor sample, were lipophillically dyed with DiD and incubated for 20 minutes at 37°C with a custom-made hydrogel slide. Unbound cells were washed away with warm X- VIVO. CD8^+^ T cells (shown stained red) are visible at the single cell level bound to the Flu tetramer, which is visualised by the 5% AlexaFluor532 conjugated to streptavidin (shown as a green spot), on the ProScanArray. Composites show the co-localisation of Flu-specific CD8^+^ T cells to the Flu M1 tetramer spot. Few if any T cells are bound to the CMV pp65 pMHC spot or the negative control random pMHC library (also tetramerised with 5% AF532-streptavidin in streptavidin). Limits of detection in both assays were ≤0.1% of the CD8^+^ population.

**Fig 4:**
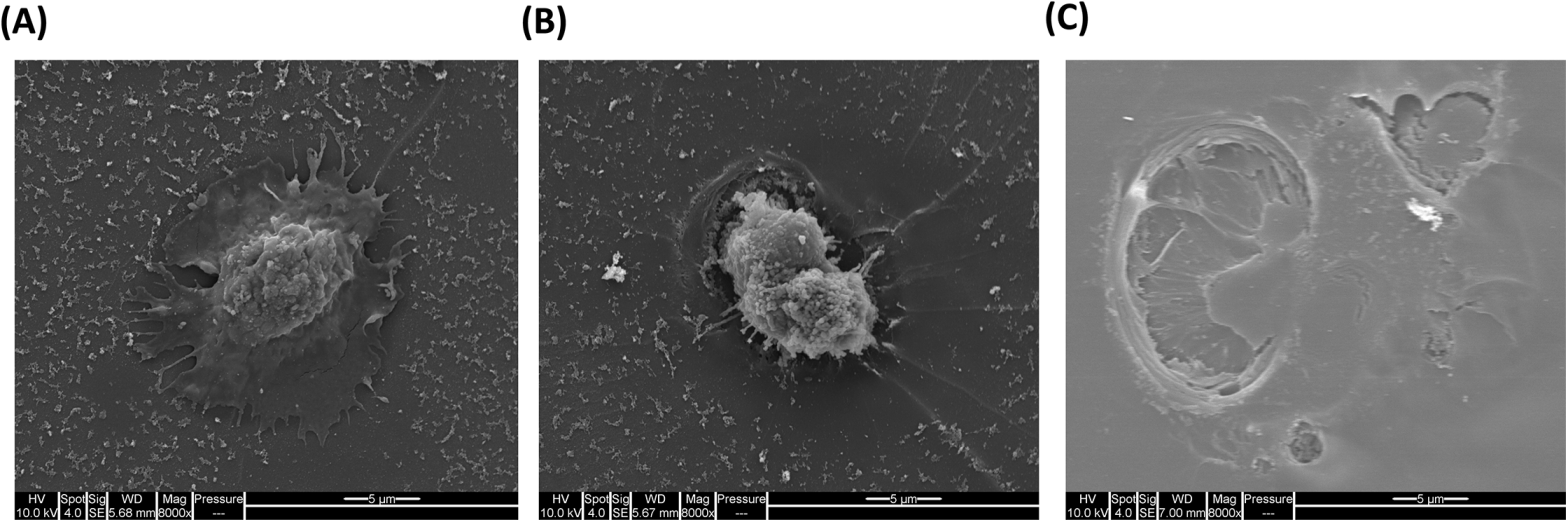
CD8^+^ T cells from leukaemia patients binding to pMHC spots on the pMHC array. **(A + B)** SEM showing a CD8^+^ positive T cell bound at the site of a tetramer spot on the pMHC array; **(C)** a single pit on the pMHC array suggesting that T cells dig into the hydrogel when binding to pMHCs and subsequently detach.

To demonstrate the robustness of the pMHC array in our hands we analysed CD8+ T cells isolated from the peripheral blood of five normal donor samples of which three were HLA-A*0201 positive and had known CMV status (as determined by serology) and Flu-specific T cells as determined by flow cytometry. By flow cytometry we could reproducibly detect viral antigen-specific T cells that recognised CMV pp65 and Flu M1 epitopes at frequencies of less than 0.02% within the total purified CD8^+^ population. This was achieved using less than 2 x 10^5^ CD8^+^ T cells per test and collecting ≥80,000 events (exemplified in **Fig 3A**). In the same samples we could detect CMV pp65 and Flu M1 specific T cells at the same sensitivities using the pMHC array (**Fig 3B**). CD8^+^ T cells bound to the array were magnified using SEM, which indicated that CD8^+^ T cells were bound at the site of the pMHC spot. Furthermore, they showed a phenotype indicative of activation (**Fig 4A & 4B**) possibly caused through their interaction with a high density of pMHCs on the flat array surface. In addition pits were observed (**Fig 4C**) which appeared to be where T cells had been on the gel surface and then detached. Previous studies [48] have shown that T cells can internalise pMHCs and this may have caused the release of T cells from the hydrogel surface and an underestimation of T-cell binding to the pMHC spots.

### Identification of simultaneous T cell populations in leukaemia patients

**Fig 5:**
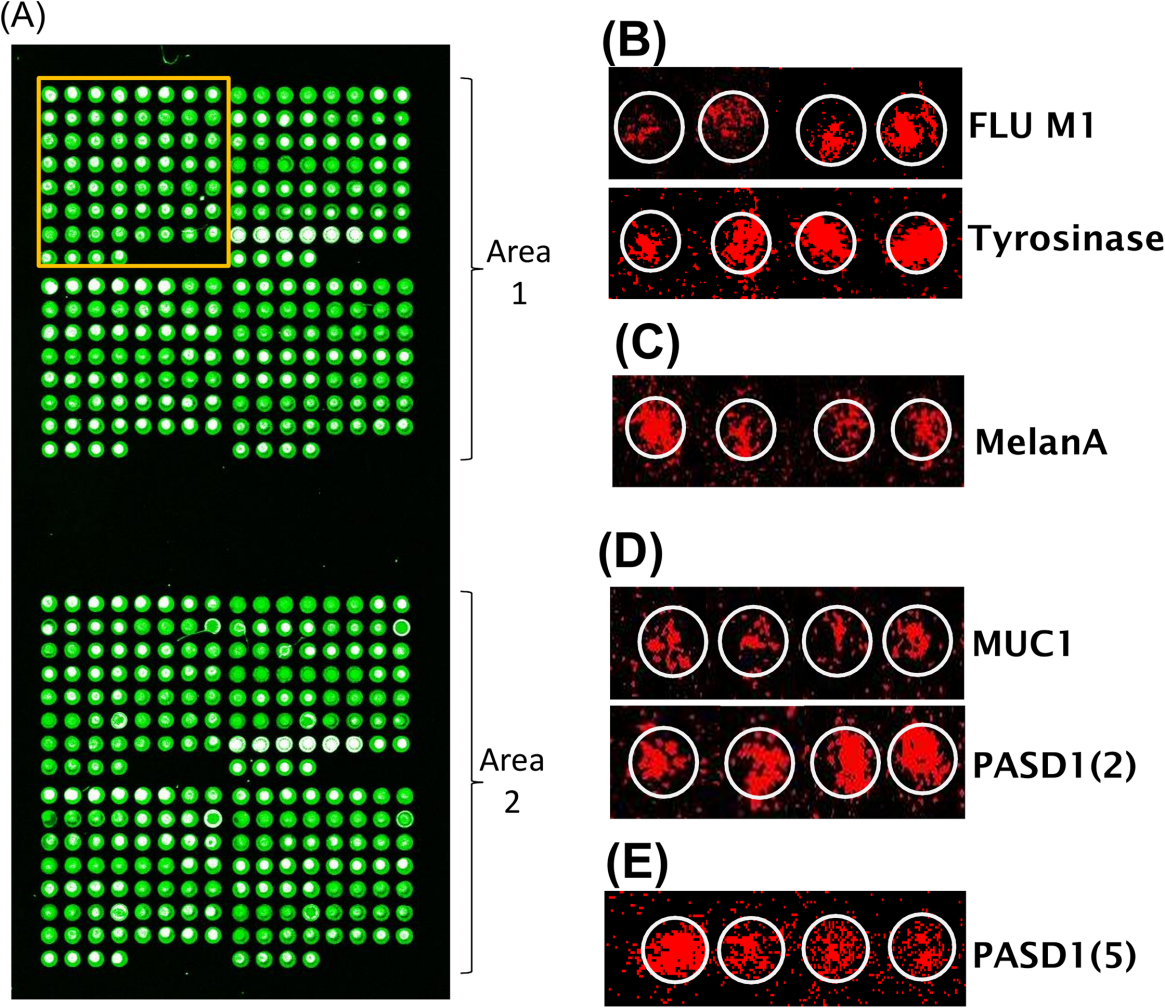
Analysis of leukaemia patient samples on the pMHC array. pMHC array (A) printed with 40 tetramers visualised by AlexFluor532 (shown in green, with bleaching to white at the highest intensity). Each pMHC complex were printed six times consecutively on one or two rows in two different areas of the hydrogel; Each line contains up to seven pMHC spots shown within the orange box; (B-E) white circles indicate the position of the pMHC spot on the array while CD8^+^ lymphocytes visualised by DiD lipophilic staining (shown in red), indicate the presence of epitope specific T cells recognising (B) FLU M1 and Tyrosinase in AML016; (C) MelanA in AML013FU; (D) MUC1 and PASD1(2) in ALL003 and (E) PASD1(5) in AML001. All epitopes described were conjugated to MHC Class I (HLA-A2) complex.

We examined the simultaneous recognition of 40 viral and leukaemia-associated tetramers (**Fig 5A; Table 1**) by negatively purified CD8^+^ T cells from the peripheral blood of 11 leukaemia patients. Approximately 45% of Caucasians are HLA-A2 positive [49] and in the cohort of AML patients studied here, (12/26) 46% were HLA-A2 positive. We did not record ethnicity for the purposes of this study. It is notable that one patient became HLA-A2 positive post-transplant (AML022-FU), We found only 4/12 of the AML patients were known to be CMV sero-positive and three of these were found to be pMHC-HLA-A2/CMV pp65 positive, two of which were also CMV IE1 positive (Table 2). Only one of the six ALL patient samples had CD8+ T cells which recognised pMHC-HLA-A2/CMV pp65 and IE1 reflecting an overall lack of epitope-specific T cells in the ALL and CML patient samples studied.

**Table 2:** Results from pMHC array analysis where there was detectable binding of virus and LAAs-specific pMHCs by “untouched” CD8^+^ T cells purified from leukaemia patients.

Patient	HLA-A2/CMV	HLA-A2—pMHC Molecules													
ID	status	CMV IE1	CMV pp65	Flu M1	CEAM5	G250	MelanA	MUC1_950-958_	PASD1Pa14	PASD1(1)	PASD1(2)	PASD1(5)	p68 RNA Helicase	Tyrosinase	WT1_126-134_
AML001	+/-														
AML002	+/+	+	+				+		+					+	
AML003	+^a^/+														
AML004	+/+	+	+						+					+	+
AML005	-/+														
AML006	- ^a^ /+														
AML007	-/+														
AML008	+/+		+						+						
AML008^b^	+/+														
AML009	-/nk														
AML010	A*24/nk						+								
AML011	- ^a^ /nk														
AML012	+ ^a^ /nk										+				
AML013	+/nk														
AML013^b^	+/nk						+								
AML014	+/nk										+				
AML015	+/nk														
AML015^b^	+/nk														
AML016	A*11;30/nk			+										+	
AML016^b^	A*11;30/nk														
AML017	A*01;24/nk										+				
AML017^b^	A*01;24/nk														
AML018	-/nk														
AML018^b^	-/nk														
AML019	+ ^a^ /nk														
AML020	- ^a^ /nk										+		+		
AML021	- ^a^ /nk				+										
AML022	-/nk														
AML022^b^	+ ^a^ /nk									+					
AML023	+/nk														
AML023^b^	+/nk					+									
AML024	nk/nk														
AML025	nk/nk														
AML026	nk/nk														
ALL001	+/+	+	+												
ALL002	+/nk														
ALL003	+/nk							+							
ALL004	nk/nk														
T-ALL005	+/nk														
cALL006	+/nk														
cALL007	+/nk														
CML001	+/nk														
CML002	-/nk														
CML003	-/nk														
CML004	+ ^a^ /nk														
CML005	-/nk														

^a^As determined by flow cytometry

^b^FU: follow-up samples; nk: data not known.

In contrast, fourteen AML patients recognised one or more LAA epitope, eight of these AML patient samples had T-cells which recognised the HLA-A2/PASD1 pMHCs, four AML patients recognised PASD1(2) [50], three AML patients recognised the PASD1 Pa14 [42], with one patient recognising the PASD1(5) epitope [50].

Patients which recognised one or more antigens also recognised the tyrosinase epitope [24], while one (AML002) recognised the HLA-A2/MelanA epitope [51,52] and the other (AML004) the WT1_126_ epitope (**Fig 5B–5E**)[30]. It was interesting to note that the patient with T cells that recognised HLA-A2/WT1_126_, did not recognise HLA-A2 WT1_126_ tetramers which harbour the A245V mutation. This mutation has been shown to enable binding of only the most avid TCRs, often only binding 10% of patient T cells who have specificity for the unmutated form [30]. Six known HLA-A2 positive patients recognised none of the pMHCs on the array (**Table 2**).

We agreed a strict criteria that at least three of six pMHC spots, in both areas of the pMHC array, had to be bound by epitope specific T cells in order to be scored positive. This was not the case for ALL003 who had T cells binding to two of six PASD1(2) pMHC spots in two regions of the array (**Fig 5D**) as well as MUC1-specific T cells, and AML001 who had PASD1(5)-specific T cells on two of six pMHC spots in region one and three of six spots in region two of the pMHC array (**Fig 5E**). This suggests we may have been underscoring the presence of LAA-specific T cells in patient samples but we retained a cut-off for analysis that we knew was robust from our optimisation phase of analysis using normal donor samples.

We did not know the UPN or HLA type of samples when scoring spots and this “blinded” process was used to ensure our scoring integrity. However most of the pMHCs used were HLA-A2 restricted and those that were not were predominantly EBV epitopes used to analyse a single patient at multiple time points who had post-transplant lymphoproliferative disease and EBV reactivation (Guinn and Orchard, unpublished data).

### PASD1 protein expression in patients’ samples

**Fig 6:**
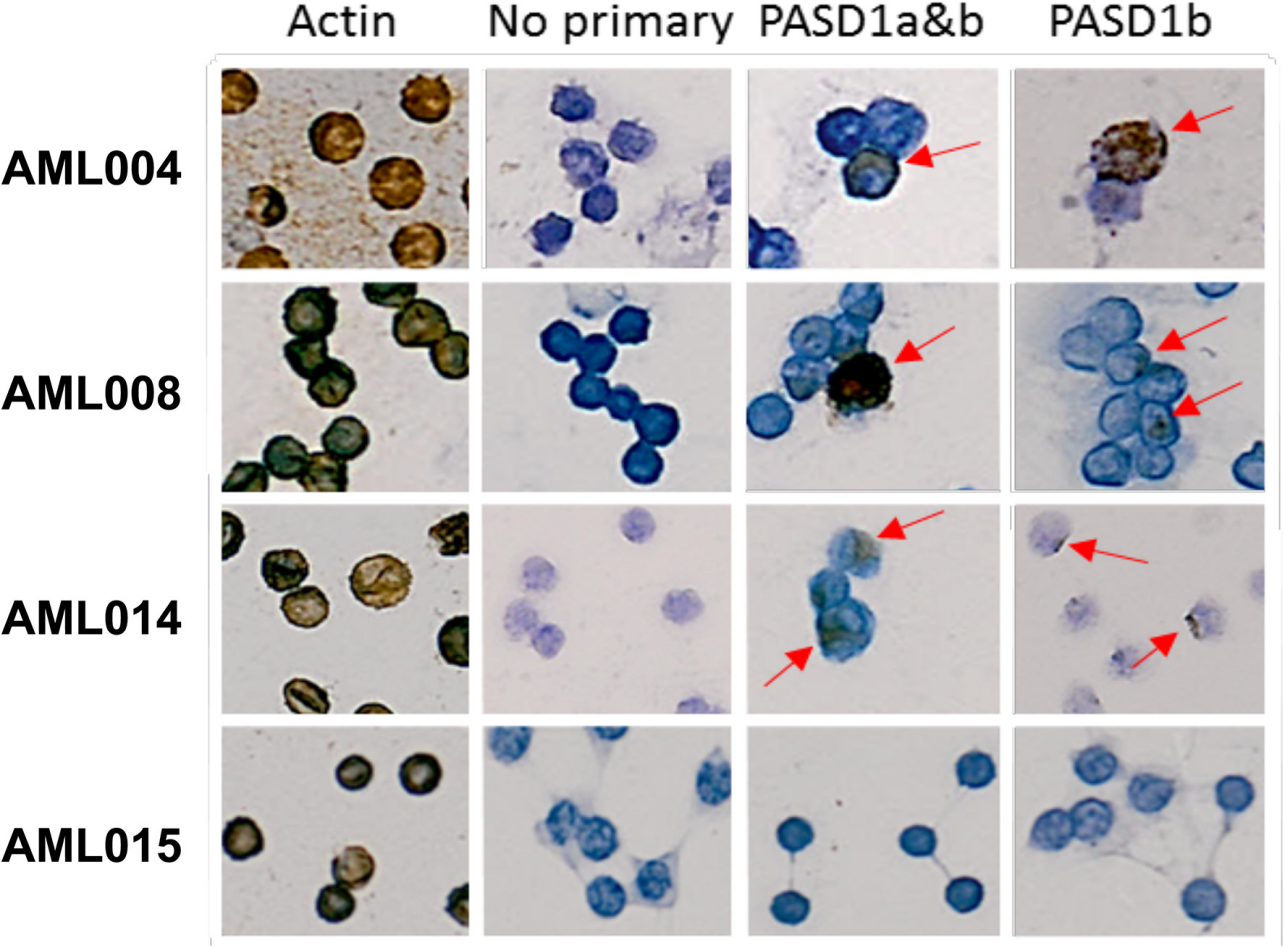
PASD1 expression in AML patient samples. Immunolabelling was used to detect PASD1 protein expression in leukaemia patient samples. Haematoxylin provides the blue background stain to allow differentiation of the cell nucleus from the cytoplasm. The brown precipitate indicates a positive reaction between secondary antibody and detection reagent, at the antigen site. In this image AML004, AML008 and AML014 immunolabelled cells are shown, AML015 was one of the samples which did not have demonstrable PASD1 expression. No primary antibody was used as a negative control and actin was used as a positive control for immunolabelling.

Using immunocytochemistry with two well-characterised PASD1 monoclonal antibodies [40] we observed PASD1a and PASD1b expression in three AML patient samples: AML004, AML008 and AML014 (**Fig 6**) but not in the other 15 patient samples tested (AML001, AML003, AML006, AML009, AML013, AML015, AML018, AML019, AML021, AML023, AML026, ALL001, ALL002, CML001 and CML002). In the three patients who had PASD1 protein expression in their peripheral blood/leukaemia cells this concurred with the presence of PASD1-specific CD8+ T cells in the periphery as detected by the pMHC array.

## Discussion

The use of pMHC technology can provide important information about the effector T-cell response implicated in tumour immunity. The approach has many benefits including the use of small numbers of T cells (0.8–1.25 x 10^6^ cells/array), minute amounts of pMHC (1ng/spot; 1/1000^th^ that required for flow cytometry) and the large numbers of spots (up to 1,000) that can be arrayed without haplotype restriction. The pMHC array requires an array printer and scanner but such equipment is available in most research institutes due to the expansion of genomic studies over the last decade. The pMHC array can also be used to detect as many specific T-cell populations as there are pMHC molecules. Of particular note the pMHC array technique is inexpensive and can be performed rapidly, making its use as a point of care predictor of response to therapy a real possibility.

In keeping with previous findings [9], our own optimisation studies demonstrated that the pMHC array can detect the same specific T-cell populations as the flow cytometer, suggesting the two techniques share comparable sensitivities. Indeed despite the small amount of pMHC molecules per spot on the array, and the low frequency of pMHC availability (estimated to be less than 2% of the total pMHC spotted)[9], the pMHC array can detect T cell populations in patients which exist at clinically-relevant frequencies (0.1% of the total CD8^+^ population), with similar sensitivity to conventional methods of pMHC staining i.e. flow cytometry. Recently Deviren et al [53] described a pMHC array method in which dimeric MHC-immunoglobulin complexes (Kb-Ig) were loaded with peptide. These Kb-Ig microarrays, using splenocytes from a C57BL/6J mouse, were demonstrated to have a lower detection limit than the conventional pMHC arrays described in these studies. The group also showed that performing the microarrays under mild shear flow condition produced more uniform distributions of captured T cells on individual spots and better spot-to-spot reproducibility across the array. Another variation of the pMHC array technique, described by Kwong et al [54], called “Nucleic Acid Cell Sorting” (NACS) involves the site-specific immobilization of pMHC tetramers by single-stranded DNA oligomers on glass slides. The authors state that the method outperforms spotted arrays in their hands when assessed on the key criteria of repeatability and homogeneity but we found neither aspect compromised in our hands due, we believe, to our aggressive washing technique and use of negatively isolated “untouched” CD8^+^ T cells. However both the Kb-Ig and NACS arrays may reduce T-cell loss, through the prevention of pMHC molecules uptake by bound T cells at room temperature [48]. Such uptake may explain the pitting we observed in the hydrogel when assessing pMHC spots using SEM (**Fig 4C**) as our arrays were incubated at 37°C for 20min prior to scanning.

The frequency of T cells adhering to a pMHC spot is believed to depend on a number of factors including the abundance of the T cells expressing the appropriate TCR, levels of TCR expression, and their affinity for the pMHC complex [9]. Development of a bound CD8+ T cell quantitation system is still required, although single cells were visible on the array at high magnification. In addition we used SEM to examine the features of T cells bound to the pMHC array. This technology did not lend itself to determining which pMHC spots had T cells bound but did demonstrate the morphology/activated nature of T cells bound to pMHC spots (**Fig 4A and 4B**). When we examined T cells following array scanning under the light microscope we found that almost all bound T cells appeared dead with a granulated nucleus and blebbed surface typical of cells undergoing apoptosis (data not shown). To minimise this we performed all steps and used solutions at 4°C, but this had little if any measurable impact on the number of T cells bound to the pMHC spots to the array, T cell survival or the frequency of array gel pitting. Activation induced cell death may have been caused by the high density of pMHC printed on the flat surface of the hydrogel, toxicity from the chemicals in the polyacrylamide gels or high temperatures and drying in the scanner. This death occurred rapidly and was seen within 25 minutes of first spotting T cells onto the pMHC arrays.

En mass production of all pMHCs for printing on the pMHC array is the most expensive and time consuming part of this technology although this may be circumvented through the use of UV-induced peptide exchange technology [55,56]. There is some limitation to the technology as it depends on the affinity of the new ligand for the MHC molecule [56,57] but studies have shown that the pMHC complex formed after UV-induced ligand exchange is identical to that obtained by classical refolding [58].

We chose to store purified negatively isolated “untouched” CD8^+^ T cells from patients in liquid nitrogen and only print pMHCs onto hydrogel slides in the week prior to analysis of groups of patient samples. Printing was performed in batches of up to 50 slides, although up to 200 can be printed in a day using the Genetix printer. Biotinylated pMHC monomers were stored at -80°C and tetramerised in the week prior to printing. One of the issues considered was the deterioration of some pMHCs after printing although Soen et al [9] suggested that the life of pMHCs are extended from one month at 4°C to six months at 4°C in hydrogel. To determine when the pMHCs had deteriorated we printed single pMHC arrays with pMHCs tetramerised from independent aliquots of monomers two weeks apart so that the time-point of deterioration of the pMHC would be obvious. We found that some pMHCs started to deteriorate in the hydrogel after six weeks, as seen by decreased levels of AF532 fluorochrome in the gel at the site of pMHC spotting and decreased T cell binding by samples. Hence we analysed the negatively purified CD8^+^ T cells on arrays within one month of printing.

PASD1 is one of the most frequently expressed tumour antigens in AML [59] and PASD1 epitopes have been found to be recognised by T cells from AML patients ([42] and this study). However this is the first study in which PASD1-specific T cell populations have been examined in direct comparison to other LAA-specific T cells in AML and PASD1 epitopes appears to be the most frequently recognised. However this may reflect the predominance of PASD1 pMHCs on the array which will be redressed in future studies.

We examined the protein expression of PASD1a and PASD1b in 18 of the patient samples in our study. Only three samples expressed both PASD1a and PASD1b protein of the eight samples which had PASD1-specific pMHCs. The lack of expression in other patient samples, despite the presence of PASD1-specific T cells in the periphery suggests that these samples had not attracted PASD1-specific T cells from the periphery to the tumour or the T cells had already killed the PASD1 expressing tumour cells. Of note was the predominance of PASD1-specific T-cell responses in 8 of 26 AML patients, perhaps reflecting the presence of four HLA-A2 restricted pMHC-PASD1 on the array, rather than the single pMHC-epitopes detecting most other tumour antigens. However PASD1 has been one of the most frequently expressed CTA in myeloid leukaemia [59] while WT1 has been found to be one of the most frequently expressed LAAs [60].

We had hoped that the pMHC array would help us prioritise PASD1 epitopes for vaccine use in clinical trials but a larger cohort of AML patients would be required. Four AML patients had T cells that recognised PASD1(2) [50] however two of these four (AML017 and AML020) were HLA-A2 negative. In addition three AML patients who were not HLA-A*0201 positive (AML016, AML017 and AML020) had LAA specific T cells which recognised PASD1(5)-HLA-A*0201 presented epitopes. This was unexpected but may reflect the promiscuity of the TCR which only recognises one or two amino acids in an epitope and as such one TCR can recognize numerous pMHC, in fact each T-cell is believed to be responsive to at least four distinct determinants within three different MHCs [61,62].

It was notable that few of the pMHCs available on the array were recognised by CML or ALL patients although numbers of patient samples from these groups were extremely small (n = 5 and n = 7, respectively). The pMHC array predominantly harboured HLA-A2 restricted pMHCs, representing those most frequently requested by collaborators of Professor HG Rammensee, and all had been functionally verified through local use prior to use on the pMHC array.

To optimise the targeting of AML patient samples, when diseased cells are heterogeneous in their expression of LAAs, the targeting of multiple antigens and/or multiple epitopes therein may be more effective at removing residual disease and escape variants [63]. Synthetic long overlapping peptides have been shown to be very effective for this mode of immunotherapy [64] and can overcome some of the short-term responses seen with single peptide vaccines [65]. The fact that four patients who had PASD1 specific T cell populations also had PASD1 expression in their leukaemia cells suggests the LAA specific-T cells are present in the periphery but are not effectively killing PASD1^+^ tumour cells.

We [66] and others [67] have previously shown that the expression of above median levels of LAAs, such as SSX2IP alone, or in combination with SURVIVIN and receptor for hyaluronan-mediated motility (RHAMM), in presentation AML patients who lack cytogenetic rearrangements, can predict survival following conventional treatment (chemotherapy). This is believed to be due to the expression of distinct LAAs on leukemic blasts leading to the eradication of residual disease after intensive chemotherapy [67]. The effective clearance of dead and dying leukaemia cells by phagocytes post-treatment and the presentation of antigens in the presence of “danger signals” such as inflammation may stimulate effective anti-leukaemia T-cell responses. Indeed the reduced survival observed in older patients following conventional treatment may in part be explained by the lower LAA expression observed in these patients [67] who often respond poorly to chemotherapy. Other groups have shown a correlation between tumour infiltrating T cells and good survival in patients with oropharyngeal cancer [68]. We found no correlation between HLA-A*0201 positive leukaemia patients with T cells which recognised LAA-specific epitopes and better survival following standard therapy, than survival in those that did not have LAA-specific T cells, in part reflecting the small numbers of HLA-A2 patients analysed to date.

We have shown that our pMHC array can be used to detect CD8^+^ T cell populations which exist in leukaemia patients at disease presentation without the need for any *ex vivo* expansion. This technology may be used to track the waxing and waning of specific-T cell populations during conventional and immunotherapy treatments. In addition the simultaneous examination of LAA-specific T-cell populations will allow the examination of epitope spreading following conventional and immunotherapy treatments as well as aiding immunotherapy (timing, dose response) and personalized therapy development in the future.

## Supporting Information

S1 Table (PDF)

## Abbreviations

ALL: acute lymphocytic leukaemia
AML: acute myeloid leukaemia
CML: chronic myeloid leukaemia
CMV: cytomegalovirus
CT: cancer-testis
CTL: cytotoxic T-lypmohocyte
FACS: fluorescence activated cell sorting
FITC: Fluorescein isothiocyanate
Flu: influenza
HLA: human leukocyte antigen
LAAs: leukaemia associated antigens
NACS: Nucleic Acid Cell Sorting
PASD1: Per Arnt Sim domain 1
pMHC: peptide-MHC
SA-PE: streptavidin-phycoerythrin
SEM: scanning electron microscopy
TCR: T-cell receptor
WT1: Wilms’ Tumour gene 1
