# Supplementary material for: Application of the pMHC Array to Characterise Tumour Antigen Specific T Cell Populations in Leukaemia Patients at Disease Diagnosis
Source: PLoS One. 2015 Oct 22;10(10):e0140483. doi: 10.1371/journal.pone.0140483 (PMC4619595; doi:10.1371/journal.pone.0140483)
Supplement: S1 Table — (PDF) [file pone.0140483.s001.pdf]

Patient ID	Age [a]	Disease [b]	Comments	Full Haplotype	WBC count [c]	Cytogenetics	FLT3 and NPM status	Survival in months (status)	Positive for TAA-specific T cells on the pMHC array (& detection of PASD1 by ICC)
AML001	59	AML (M2)	initial chem, failed to achieve CR with induction<br>VUD allo; ATRA chemotherapy initially, as suspicion of APML; Admitted for ADE chemotherapy x 1 cycle; Admitted for Salvage Chemotherapy : FLA-IDA<br>Allo tx	**A*02,** B*08;40, Cw*03,07<br>DRB1*0401, 0301, DQB1*0301, 0201/02	30.4	Normal	FLT3 and NPM1 wt	59 (D)	-(-)
AML002	65	AML	chemotherapy x? CR; Cycle 1 & 2 of DA chemotherapy; 4 cycles of MIDAC chemotherapy; Relapsed. Treated with DA 3+10. Followed by TaNK trial at Royal Free Hospital. Further relapse.	**A2**	12.0	Not tested	NK	29 (D)	+ (ND)
AML003	48	AML	chemotherapy x4 CR; AML15 trial patient randomized to: 2 x DA cycle ; High dose Ara-C & Mylotarg:<br>HDAC cycle 4	**A2**	2.4	46, XX, many cells showed chromosome damage, ?artefact	FLT3 wt	49 (A)	-(-)
AML004	46	AML	chemotherapy x4 CR; AML15 trial patient randomized to:<br>2 x DA cycle:; MACE cycle 3: MIDAC cycle 4.	**A2**	6.0	Del 7q	FLT3 wt	14 (A)	+(+)
AML005	62	AML	chemotherapy x4 CR; AML 16 patient; DA 3+10 Cycle 1; DA 3+8 Cycle 2; MIDAC (modified) Cycle 3; MIDAC (modified) cycle 4; Completed.	A3	19.2	At diagnosis:<br>12p minus	NK	4 (D)	-(ND)
AML006	68	AML (M4)	chemotherapy x1 ; PR ; DA 1 x cycle	ND	176.1	Normal	ND	2 (D)	-(-)
AML007	64	AML	preceding MDS (CMML); chemotherapy x1 PR; DA & Cytarabine; Palliative chemotherapy : FLA	A3	31.8	ND	NK	2 (D)	-(ND)
AML008	50	AML	HDAC chemotherapy	**A02**,02;B*07,37;Cw*06,07<br>DRB1*0802,1501;DQB1*0302,0602	0.6	ND	NK	42 (D)	+(+)
	30	AML	3xcycles ; DA & Ara-C 2 cycles ; cycle 3 : FLA-IDA	A1,24;B*08,15;C*03,07	5.8	Other CG normal	FLT3	66(A)	-(-)

AML009		(M4 FAB)	Sib-allo	DRB1*0301;DQB10603,0201			ITD Positive		
AML010	30	AML	Idarubicin & ATRA<br>ATRA maintenance<br>chemotherapy only	A*24,*26;B*13,*38;<br>Cw*06,*12<br>DRB1*04,*07;DQB1*02,*03	2.9	t(15;17)	AML M3 with PML-RARA translocation	83 (A)	+(ND)
AML011	63	AML	AML 16 (2 cycles ofDA+ATRA)	NK	NK	Trisomy 13	NK	20 (A)	-(ND)
AML012	71	AML	Low dose cytarabine	NK	NK	NK	NK	50 (A)	+(ND)
AML013	45	AML	AML 17 (2 cycles of ADE, 2 cycles of HDAC)	A*02, 03	NK	Normal	NK	43 (A)	+(-)
AML014	57	MDS/ AML	ADE & mylotarg<br>MUD allo (Flu/Bu/Campath)<br>GvHD	**A*02,**03, B*38, 40, Cw*03,12, DRB1*03;01, 13:01, DQB1*02:01, 06:03	NK	Trisomy 8<br>Abnomal 13	NK	8 (D)	+(+)
AML015	19	AML	AML 17	A*01,02	NK	Normal	FLT3 wt	51 (A)	-(-)
AML016	26	AML	Chemotherapy and then sibling allograft	A*11;30	NK	NK	MLL rearrangement	25 (D)	+(ND)
AML017	59	AML	AML17 (2 cycles of Dax, MiDAC)	A*01,24	NK	NK	FLT3 wt	50 (A)	+(ND)
AML018	NK	AML	VR<br>2 x allotransplants, alive 100d after the 2nd transplant	A*11, B*07, 35, Cw*04,07, DRB1*01:01, 15:01; DQB1*05:01, 06;02	NK	Normal	NK	64 (A)	-(-)
AML019	65	AML	AML 16 (3 cycles DA)	NK	NK	Normal	NPM1	49 (A)	-(-)
AML020	54	AML	Dax2, HDAC, MiDAC	A*01:01; B*08:01:01; C*07:01, DRB1*03:01, DQB1*02:01	NK	Normal	FLT3 +<br>NPM1+	23 (D)	+(ND)
AML021	82	AML	Low dose cytarabine	NK	NK	Normal		4 (D)	-(-)

AML022	64	AML	DA x2	A*03;11	NK	Normal	FLT3 ITD +	7 (D)	+(ND)
AML023	54	MDS	VUD allo	**A*02;**03	NK	Normal	NK	7 (D)	+(-)
AML024	62	AML/ MDS	AML 16 trial (3 cycles DA,& 1 cycle Mylotarg)	NK	NK	46, XX	NK	6 (D)	-(ND)
AML025	77	AML	LDAC	NK	NK	Normal	NK	34 (D)	-(ND)
AML026	62	AML	AML 16 (3 cycles DA)	NK	NK	46 XX	FLT3 wt<br><br>NPM1+	41 (A)	-(-)
ALL001	22	ALL	Chemotherapy (UKALL 2003)	**A2**	50.4	46XY	(SET/CAN fusion transcript)	38 (A)	-(-)
ALL002	65	ALL	Chemotherapy	**A2**	230.0	t(4;11)	NK	3 (D)	-(-)
ALL003	NK	ALL	NK	NK	NK	ND	NK	NK	+(ND)
ALL004	NK	ALL	NK	NK	NK	ND	NK	NK	-(ND)
ALL005	22	T-ALL	UKALL 2003	**A*02,**03; B*35, 44; C*04,05; DRB1*14,15, DQB1*05, 06	NK	t(1;7)	NK	2 (A)	-(ND)
ALL006	50	cALL	UKALL XII; VUD allo	A*02 B*15, 57 Cw*03, 06: DRB1*01:01, 14:01, DQB1*05:01, 0503	NK	Normal	None	17 (A)	-(ND)
ALL007	26	cALL	UKALL XII	A*01,02; B*07, 13;Cw*06, 07 DRB1*15:01, 07:01; DQB1*06:02, 02;02	NK	t(1;19)	NK	16 (A)	-(ND)
CML001	67	CML	CP on Glivec, MMR	**A2 (by FACs)**	150.0	t(9;22)	NK	41 (A)	-(-)
CML002	21	CML	Imatinib, MMR	NK	NK	t(9;22)	NK	16 (A)	-(-)
CML003	63	CML	VUD allograft (poorly responsive to TKIs)	A*24, 31 B*40, 57 Cw*03, 06 DRB1*04:04, 07:01 DQB1*03:03, 03:02	NK	t(9;22)	NK	23 (A)	-(ND)
CML004	32	CML	Imatinib, MMR	NK	NK	t(9;22)	NK	27 (A)	-(ND)

CML005	61	CML – CP2[d]	Glivec ; Myeloid transformation; chemotherapy x 2 followed by an allo-transplant	A*03,11;B*15,44;Cw*03,05DRB1*0101,0401;DQB10302,0501	210.0	t(9;22)	NK	48 (A)	-(ND)

[a]Age at diagnosis

[b]Diagnosis at time of sampling

[c]White blood cell count at time of sampling x 10^9/L

[d]Myeloid blast transformation

ADE: induction therapy consisting of cytarabine, daunorubicin and etoposide; ALL: acute lymphocytic leukaemia; allo: allograft; AML: acute myeloid leukaemia; APML: acute promyelocytic leukaemia; Ara-C: Arabinosylcytosine; ATRA: all trans retinoic acid; CML: chronic myeloid leukaemia, CMML: chronic myelomonocytic leukaemia; CP: chronic phase; CR: complete remission; DA: daunorubicin and Arabinosylcytosine; FLA: chemotherapy using fludarabine and cytarabine; HDAC: histone deacetylase inhibitors; IDA: chemotherapy protocol using idarubicin; FLT3-ITD: FLT3 – internal tandem repeat; Glivec: contains imatinib and inhibits tyrosine kinases which contribute to disease, in this case inhibiting BCR-ABL activity in CML; LDAC: low dose cytarabine; MACE: chemotherapy consisting of amsacrine, cytarabine and etoposide; MDS: myelodysplastic syndrome; MIDAC: chemotherapy consisting of mitoxantrone and cytarabine; MUD: matched unrelated donor; ND: not done; NK: not known; NPM1: Nucleophosmin gene; PR: partial remission; TKI: tyrosine kinase inhibitors; transl: translocation; VUD: Volunteer unrelated donor; wt: wild type
